# Digital Training Program for Line Managers (Managing Minds at Work): Protocol for a Feasibility Pilot Cluster Randomized Controlled Trial

**DOI:** 10.2196/48758

**Published:** 2023-10-24

**Authors:** Louise Thomson, Juliet Hassard, Alexandra Frost, Craig Bartle, Joanna Yarker, Fehmidah Munir, Richard Kneller, Steven Marwaha, Guy Daly, Sean Russell, Caroline Meyer, Benjamin Vaughan, Kristina Newman, Holly Blake

**Affiliations:** 1 School of Medicine University of Nottingham Nottingham United Kingdom; 2 Queen's University Belfast Belfast United Kingdom; 3 Institute of Mental Health Nottinghamshire Healthcare NHS Foundation Trust Nottingham United Kingdom; 4 Department of Organisational Psychology Birkbeck College, University of London London United Kingdom; 5 Affinity Health at Work London United Kingdom; 6 School of Sport, Exercise and Health Sciences Loughborough University Loughborough United Kingdom; 7 School of Economics University of Nottingham Nottingham United Kingdom; 8 Institute for Mental Health, School of Psychology University of Birmingham Birmingham United Kingdom; 9 Specialist Mood Disorders Clinic, The Zinnia Centre Birmingham and Solihull Mental Health NHS Foundation Trust Birmingham United Kingdom; 10 Office of the Provost British University in Egypt Cairo Egypt; 11 Faculty of Health and Life Sciences Coventry University Coventry United Kingdom; 12 WMG, University of Warwick Warwick United Kingdom; 13 Psychology Department Nottingham Trent University Nottingham United Kingdom; 14 School of Health Sciences University of Nottingham Nottingham United Kingdom; 15 NIHR Nottingham Biomedical Research Centre Nottingham United Kingdom

**Keywords:** acceptability, anxiety, burnout, cluster randomised control trial, depression, digital training, feasibility, intervention, intervention, managers, mental health, stress, usability, work, workplace

## Abstract

**Background:**

Mental health problems affect 1 in 6 workers annually and are one of the leading causes of sickness absence, with stress, anxiety, and depression being responsible for half of all working days lost in the United Kingdom. Primary interventions with a preventative focus are widely acknowledged as the priority for workplace mental health interventions. Line managers hold a primary role in preventing poor mental health within the workplace and, therefore, need to be equipped with the skills and knowledge to effectively carry out this role. However, most previous intervention studies have directly focused on increasing line managers’ understanding and awareness of mental health rather than giving them the skills and competencies to take a proactive preventative approach in how they manage and design work. The Managing Minds at Work (MMW) digital training intervention was collaboratively designed to address this gap. The intervention aims to increase line managers’ knowledge and confidence in preventing work-related stress and promoting mental health at work. It consists of 5 modules providing evidence-based interactive content on looking after your mental health, designing and managing work to promote mental well-being, management competencies that prevent work-related stress, developing a psychologically safe workplace, and having conversations about mental health at work.

**Objective:**

The primary aim of this study is to pilot and feasibility test MMW, a digital training intervention for line managers.

**Methods:**

We use a cluster randomized controlled trial design consisting of 2 arms, the intervention arm and a 3-month waitlist control, in this multicenter feasibility pilot study. Line managers in the intervention arm will complete a baseline questionnaire at screening, immediately post intervention (approximately 6 weeks after baseline), and at 3- and 6-month follow-ups. Line managers in the control arm will complete an initial baseline questionnaire, repeated after 3 months on the waitlist. They will then be granted access to the MMW intervention, following which they will complete the questionnaire post intervention. The direct reports of the line managers in both arms of the trial will also be invited to take part by completing questionnaires at baseline and follow-up. As a feasibility pilot study, a formal sample size is not required. A minimum of 8 clusters (randomized into 2 groups of 4) will be sought to inform a future trial from work organizations of different types and sectors.

**Results:**

Recruitment for the study closed in January 2022. Overall, 24 organizations and 224 line managers have been recruited. Data analysis was finished in August 2023.

**Conclusions:**

The results from this feasibility study will provide insight into the usability and acceptability of the MMW intervention and its potential for improving line manager outcomes and those of their direct reports. These results will inform the development of subsequent trials.

**Trial Registration:**

ClinicalTrials.gov NCT05154019; https://clinicaltrials.gov/study/NCT05154019

**International Registered Report Identifier (IRRID):**

DERR1-10.2196/48758

## Introduction

### Background

Common mental health problems (eg, stress, anxiety, and depression) were estimated to account for the majority of days lost due to work-related ill health in the United Kingdom in the year 2021 to 2022 (7.0 million and 7.3 million, respectively) [1]. The annual cost of absenteeism, presenteeism, and turnover associated with mental ill health in the United Kingdom has increased by a quarter since 2019 and is now estimated to be £53-£56 (US $67.51-$71.33) billion [2]. Despite these costs, many employers are unaware of the actions they could take to support their employees’ mental health and of the potential return on investment of workplace mental health and well-being practices. Recent estimates suggest a return, on average, of £5.30 (US $6.75) for every £1 (US $1.27) invested [2].

There are 3 main categories of interventions that aim to mitigate the impact of poor mental health in the workplace. Primary interventions are preventative in focus and aim to remove or minimize work stressors (eg, excessive job demands, time pressure, and bullying at work) associated with work-related stress and poor mental health at work. Primary prevention involves changing the way that work is designed and managed to reduce the risks to employees’ work-related stress and mental health. Secondary interventions target the identification of employees already experiencing work-related stress and poor mental health through raising awareness and knowledge of common mental health symptoms and associated workplace behaviors, such as problems concentrating, feeling uptight, distressed, and fatigued [3], and aim to provide the skills and resources to bolster coping for those employees affected. Finally, tertiary interventions aim to provide treatment of symptoms, for example, return-to-work programs, employee assistant programs, occupational therapy, and medical stress interventions [3], to minimize any effects of poor mental health at work and support the return-to-work and retention of an employee. While employers should incorporate all 3 types of intervention, a primary preventative approach is widely acknowledged as the priority [4-6].

Line managers have a key role in the operationalization and impact of such preventative approaches in the workplace. They play a vital role in designing jobs, managing work tasks and workload, treating employees with respect and clarity, creating a work environment that is supportive and psychologically safe, and encouraging open and honest conversations about mental well-being at work [7]. However, line managers need to be equipped with appropriate skills and knowledge to effectively fulfill this role.

Limited mental health or stress prevention training interventions targeting line managers exist, with only a small number using a randomized trial methodology to evaluate their impact [8-10]. These intervention studies have demonstrated effectiveness in improving line managers’ knowledge of mental health [8], their skills in communicating about mental health and related resources [9], their confidence in creating a mentally healthy workplace [11], and lowered sickness absence of the employees they supervise [10]. Most of these line manager training packages have focused exclusively on increasing their awareness and knowledge of mental health at work and providing guidance on how to support and signpost employees who are already experiencing poor mental health. However, few line manager training interventions exist that aim to improve the behavioral competencies and skills of managers in preventing work-related stress and promoting mental health at work.

Equipping line managers to enable primary preventative approaches is a critical next step. Stansfeld et al’s [12] study assessed the effectiveness of an evidence-based e-learning package that aimed to improve line managers’ competencies in preventing work-related stress [13,14]. However, this study only used employee-related outcome measures of psychological distress and sickness absence, which remained unchanged, and did not examine changes in line managers’ confidence, knowledge, or behavior.

This study takes a preventative approach by training line managers but broadens the content of the training beyond that of Stansfeld et al’s [12] intervention to include multiple components (based on evidence and theory-driven) on the causes of work-related stress and poor mental health at work. The development of the MMW digital learning intervention is fully described in Blake et al’s paper [15].

In terms of the outcome measures studied in trials to date, most studies have used primary outcomes relating to line managers (such as changes in their confidence and knowledge about mental health), which are closely linked to the content and purpose of the training. A small number of studies have explored employee outcomes such as well-being and sickness absence [12], employee distress [11], employees’ perceptions of changes in their manager’s behavior [9,11], and employees’ use of resources available [9]. This study will build on the methods adopted by others to consider the feasibility of collecting data on the outcomes more closely related to digital training for line managers as well as outcomes related to employees, specifically their well-being and productivity.

The delivery of the training for line managers in previous trials has largely been based on face-to-face sessions [8-10] although 2 studies used web-based delivery [11,12]. With a rapid increase in the use of web-based workplace training due to the COVID-19 pandemic, the use of e-learning and digital training packages has been embraced by many employers as a way of offering more flexible learning to employees [16] and is advocated to be cost-effective as a delivery method [17]. Furthermore, providing more control to individual learners may increase motivation for, and satisfaction with, learning [18,19]. Therefore, the evaluation of digital learning and training resources has relevance in the modern workplace, and studies are needed that explore their use and acceptance by learners when related to workplace mental health. This study will primarily use qualitative data to explore the usability and acceptability of the digital mode of training and how this could be further enhanced to increase engagement and support the transfer of learning into practice.

### Study Aims and Objectives

#### Aims

This study is part of a larger research program, the Mental Health and Productivity Pilot (MHPP) which is funded by Midlands Engine [20]. The broader aims of the MHPP program are (1) to reduce the impact of poor mental health in the workplace and barriers to employability and productivity; (2) to reduce stigma around workplace mental health; and (3) to deliver evidence-based, locally relevant, tested, and sustainable workplace programs to suit the needs of employers and employees.

This study describes a pilot and feasibility trial of a digital intervention with interactive training materials delivered on the internet to line managers (ClinicalTrials.gov NCT05154019). The intervention aims to increase managers’ confidence, knowledge, and behavioral competencies to prevent work-related stress and promote mental health at work among the people they manage. The MMW training for line managers includes existing evidence-based resources that support line managers in reducing work-related stress and developing a workplace that actively promotes and supports the mental health and well-being of the people they manage (their direct reports). This study aims to determine the feasibility of a full cluster randomized controlled trial (RCT), testing the effectiveness of the MMW training on improving line managers’ outcomes (confidence, knowledge, and behavior) and direct report outcomes (well-being, perceptions of line manager behaviors, and sickness absence).

#### Objectives

To assess the feasibility of a full trial of the MMW intervention, our specific objectives are to test:

1. The potential for uptake within small, medium, and large organizations by identifying and monitoring the:
  willingness of employers to register interest in participating in the trial and allow line managers to take part in the trial;
  recruitment and retention rates of line managers in the trial; and
  recruitment and retention of managers’ direct reports.
2. The perceived suitability and effectiveness of the MMW intervention for line manager training by determining the:
  acceptability, usability, and utility of the training among line managers and stakeholders within the participating organizations;
  potential for improving line managers’ confidence (primary outcome), knowledge, and behaviors (secondary outcomes) to inform the planning of a larger trial;
  potential for improving direct report outcomes (well-being, absence, and self-reported productivity) by assessing changes to inform the planning of a larger trial; and
  barriers and facilitators to the intervention implementation and effectiveness.
3. The data collection methods for primary and secondary outcome measures are to:
  inform key parameters for a larger trial relating to sample size and clustering.

How these research objectives map onto the data collection methods and measures is depicted below (Table 1).

**Table 1:** Mapping of research objectives onto data collection methods and measures.

Research objective	Method of data collection	Data source or measure
Willingness of organizations to take part in trial	Study records Interviews with stakeholders	% conversion from interest to participating Qualitative data
Acceptability of waitlist control	Interviews with stakeholders Researcher diaries	Qualitative data
Willingness to allow access to organizational level data on absence	Interviews with stakeholders Researcher diaries	Qualitative data
Recruitment of line managers	Study records	% eligible line managers who consent
Retention of line managers	Web-based survey data	% consenting managers who complete follow-up
Recruitment of managers’ direct reports	Study records	% eligible direct reports who consent
Retention of managers’ direct reports	Web-based survey data	% consenting direct reports who complete follow-up
Acceptability, usability, and utility of the intervention	Interviews with managers and stakeholders End of module feedback from line managers	Qualitative data Qualitative feedback and quantitative data: fidelity delivery and engagement, implementation qualities [21]
Barriers and facilitators to the intervention implementation and effectiveness	Interviews with line managers and stakeholders End of module feedback from line managers	Qualitative data Qualitative feedback
Improvement in line manager confidence	Web-based survey	Confidence to create a mentally healthy workplace [11,22]
Improvement in line manager mental health knowledge	Web-based survey	Mental Health Knowledge Scale [23] Mental Health Literacy in the Workplace [24]
Improvements in line manager ratings of their managerial behavior	Web-based survey	Management Competency Indicator Tool-manager version [13]
Improvement in direct reports’ well-being	Web-based survey	Warwick-Edinburgh Mental Well-Being Scale 14-item [25]
Improvements in ratings of manager behavior	Web-based survey	Management Competency Indicator Tool-employee version [13]
Improvement in direct reports’ sickness absence and productivity	Organizational records Self-reports	Sickness absence rates Productivity rating

## Methods

### Ethics Approval

The study will be conducted in accordance with the ethical principles that have their origins in the Declaration of Helsinki, 1996; the Principles of Good Clinical Practice; and the UK Department of Health Policy Framework for Health and Social Care, 2017. Ethics approval has been granted by the University of Nottingham’s Faculty of Medicine and Health Sciences Ethics Committee (Ref. FMHS 299-0621, with amendment approved June 6, 2022). No recruitment, data collection, or intervention delivery will take place before ethical approval is in place. Informed consent will be collected for each participant in the study, following the provision of detailed information about the study. This will emphasize that participation in the study is entirely voluntary, and participants are free to withdraw at any time. Participants will not be compensated for taking part in the study. All data are to be stored securely in password-protected storage with any identifiers removed in adherence to the General Data Protection Regulations (GDPR, 2018). Any quotes from interview participants used in reports and articles will be anonymized.

### Design

This feasibility pilot study is a multisite, 2-armed cluster RCT taking place in work organizations of different sizes and sectors across the Midlands area of England. The organizations are the units of randomization (the clusters), with data collected from individual line managers (the participants) and the direct reports who are supervised by managers within those organizations. If large, multisite organizations are recruited for the study, we will consider the option of using an internal control group within that organization if there is a low risk of contamination between the intervention arm and control arm within that large organization. The data collection schedule for the study is summarized below for the intervention arm (Table 2) and the control arm (Table 3). A repeated measures design will be adopted, whereby participating line managers in the intervention arm will complete web-based questionnaires at baseline, on completion of the MMW training (approximately 6 weeks after baseline), and at 3- and 6-month follow-up periods after baseline. A 3-month waitlist control group will be used with participating line managers. They will be asked to complete web-based questionnaires at baseline, 3-month follow-up (before starting the intervention), and after completion of the training (6 months after baseline).

**Table 2:** Data collection schedule for the intervention arm.

			Baseline survey		Postintervention survey (approximately 6 weeks after baseline)		3 months after the baseline follow-up survey		6 months after the baseline follow-up survey		Process evaluation interviews
**Intervention arm**											
	Managers	✓		✓		✓		✓		✓	
	Direct reports	✓				✓		✓			
	Stakeholders									✓	

**Table 3:** Data collection schedule for the control arm.

			Baseline survey	Preintervention survey (3 months after baseline)		Postintervention follow-up survey (6 months after baseline)		Process evaluation interviews
**Control arm**								
	Managers	✓		✓	✓			
	Direct reports	✓		✓	✓			
	Stakeholders						✓	

### Setting

The study setting will be any organization with line managers and their direct reports within the Midlands area of the United Kingdom.

### Sample Size

A minimum of 8 clusters (randomized into 2 groups of 4) will be sought to inform a future trial, as recommended for pilot clinical trials [26]. We will aim to recruit 30 line managers for each arm as per guidance for feasibility trials [27].

### Recruitment

#### Organizations

Participating organizations will be recruited through the communication channels (Twitter, newsletters, and website) related to the wider MHPP program. A flyer advertising the study will inform organizations about the aims of the study and ask them to register their interest in participating through a web-based form. Organizations that register their interest will receive a follow-up phone call from a study researcher to provide further information about the study. We will identify the “gatekeeper,” the person within the organization who will support the implementation of the study within that organization by facilitating communication with line managers and promoting the study within the organization. We will share study details, including program content and randomization. Organizations may want to provide the intervention to managers within certain departments or divisions or at certain levels of seniority within their organization. These options will be discussed as part of the organization’s recruitment for the study to identify whether all managers or specific groups of managers will be approached for participation in the study. Once agreed upon in principle, we will request written informed organizational consent at the site level for the research.

#### Line Managers and Direct Reports

Managers within the participating organizations will be approached to participate in the study through a poster or flyer about the study, which will be shared with them by the gatekeeper within the organization. Line managers who express interest in taking part will be given information about the study and asked to provide written informed consent before participating in the study. To take part in the study, line managers must be aged 18 years or older (no maximum age but must be employed with managerial responsibilities); have direct managerial or supervision responsibility for 3 or more staff; have a work computer or mobile phone and email address with which to access the training and receive reminders; and be able to provide informed consent. Line managers will be excluded from the study if they are due to retire or be made redundant in the next 6 months or have undertaken work-based training on mental health at work within the past 6 months.

To assess any changes in outcomes of line managers’ direct reports, all participating managers will be asked to share details of the study with their direct reports to invite them to also participate in a web-based survey at at baseline, 3-month and 6-month follow up. Participant information sheets and consent forms will be provided on the internet before these surveys are completed. Direct reports will not have any access to the training intervention itself.

All participant information sheets and consent forms will be in English. As the intervention we are testing will only be delivered in English, it will not be possible to include non-English speakers in this feasibility and pilot study. It will be explained to all potential participants that entry into the study is entirely voluntary and that they can withdraw from the study at any time without negative repercussions.

### Allocation to Intervention

Organizations (clusters) will be randomly assigned (1:1) to either the intervention or waiting list control group using a web-based random sequence generator [28]. This allocation will be stratified to ensure a spread of different sizes of organizations within the intervention and control arms. Where the organization is sufficiently large to have distinct departments, divisions, or units with minimal risk of contamination between them, we will randomly assign 2 of these different departments, divisions, or units to either intervention or waiting list control using the same approach.

The research team and participants will not be blinded to the outcome of randomization due to practical reasons (ie, sending reminder emails to participating managers). The lead researcher will enroll participating organizations and line managers and will assign organizations (clusters) to the intervention or control arm.

### The MMW Intervention

#### Developing the MMW Intervention

**Figure 1:**
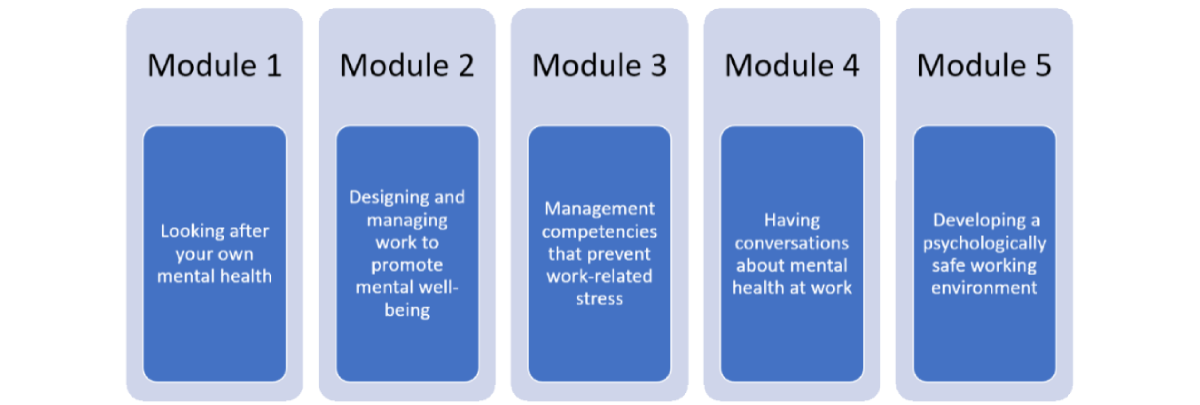
Overview of the content of the Managing Minds at Work modules.

The intervention was co-designed with stakeholders to ensure it addresses employer needs and supports the engagement of participating organizations. The intervention development process was guided by Agile Science approaches as used in 2 previously published studies by Blake and colleagues [29] and fully described in Blake et al’s study [15]. Stakeholder consultation and review was an iterative process, allowing for continuous delivery and a resource-efficient approach to training development. See Figure 1 for an overview of the MMW content. The MMW intervention materials were developed with considerable input from a range of stakeholders, including academic experts, learning and development experts, well-being managers, organizational representatives, and line managers. The MMW intervention is described throughout as a digital training course, and the modules therewithin refer to the various stages of this training.

#### The MMW Training Intervention

The intervention is a digital, interactive training course delivered on the internet that includes evidence-based material, guidance, and practical exercises. It will be made accessible to participants through a secure website using a personal computer, laptop, tablet, or mobile phone. Although larger screen devices (eg, personal computer or standard laptop) are recommended to maximize readability. Individual usernames and passwords will be provided to each participating line manager to gain access to the training. The training materials are presented using a web-based, interactive, and multimedia software called Xerte [30]. The training material includes evidence-based guidance, interactive tasks with opportunities for reflection, short videos, embedded links, and downloadable checklists (an in-depth overview of the modules, using the TiDiER [Template for Intervention Description and Replication] checklist, is described in the MMW intervention development paper) checklist [15]. The training covers 5 topic areas, presented as a series of independent, stand-alone modules:

- Module 1: looking after your own mental health
- Module 2: designing and managing work to promote mental well-being
- Module 3: management competencies that prevent work-related stress
- Module 4: developing a psychologically safe workplace
- Module 5: having conversations about mental health at work

Each module is designed to take between 20 and 30 minutes to complete. Participants will be permitted by their employer to complete the training within their work hours. The training is self-led, so each participant can progress through each module at their own pace and at a time that suits them. At the start of the intervention, a suggested schedule will be provided to each participant, whereby line managers are encouraged to schedule and complete 1 module per week. Reminder emails will be sent to participants on a weekly basis to encourage engagement and completion of that week’s module.

Organizations (clusters) will communicate with line managers (either all line managers or those in selected divisions, departments, or levels of seniority) about the intervention and study. Managers in the intervention arm will be invited to register for the training and start it right away. Managers in the control arm will be invited to register for the training and start it after a 3-month wait.

### Outcome Measures

#### Trial Feasibility and Acceptability-Related Outcomes

To address objectives 1a, 1b, 1c, 2a, and 2d, we will collect data on the following to assess the feasibility of a trial and acceptability of the intervention:

1. Number of employers registering interest in participating in the study
2. Number of employers consenting to take part in the study (minimum of 8 organizations recruited within a 6-month period)
3. Number of line managers sent information about the study
4. Recruitment of line managers into the study in both the intervention and control arms, and retention of those recruited through the follow-up points to completion in the intervention and control arms (70% at 3 months and 50% at 6 months)
5. Recruitment and retention of line managers’ direct reports
6. Line manager views on the acceptability, usability, and utility of the intervention through postmodule feedback forms and end-of-study interviews
7. Stakeholders’ views on the acceptability, usability, and utility of the intervention through interviews
8. Possible barriers and facilitators to the intervention implementation and effectiveness through interviews with line managers and stakeholders.

#### Research-Related Primary and Secondary Outcomes

In relation to objectives 2b, 2c, and 3a, we will collect the following data to assess the potential for improving line manager and direct report outcomes and inform the planning of a larger trial. The primary outcome we will assess is line managers’ confidence in creating a mentally healthy workplace [11,22,31]. The secondary outcomes will be line managers’ mental health knowledge [23]; line managers’ workplace mental health literacy [24]; line managers’ self-rating of behavior (Stress Management Competency Indicator Tool [SMCIT]–manager version) [13]; line managers’ psychological well-being (Warwick-Edinburgh Mental Well-Being Scale [WEMWBS]) [25]; direct reports’ psychological well-being (WEMWBS) [25]; direct reports’ rating of line manager behavior (SMCIT-employee version) [13]; direct reports’ sickness absence (organizational records); and direct reports’ productivity (self-reported).

### Data Collection Procedure in Intervention Arm

In the intervention arm, line managers will be invited to complete the digital training. Participants will be encouraged to complete the modules in succession, from introduction through to modules 1 to 5, aiming to complete a module a week. The training program should, therefore, be completed in 6 weeks. Data will be collected as per Table 2, at baseline, on completion of the MMW training (approximately 6 weeks after baseline), and at 3- and 6-month follow-up periods from baseline. Line managers’ direct reports will be invited to take part in the study to determine whether they notice any change in their line manager’s behavior or whether their own mental health outcomes are affected by the line manager training. Line managers’ direct reports will therefore be invited to complete web-based questionnaires at baseline when the line manager starts the training, and 3 and 6 months later. Where available, data will also be collected from organizational records on the sickness absence levels of employees managed by the participating line managers.

### Data Collection Procedure in Control Arm

Line managers in the organizations within the control arm will receive the intervention after a 3-month wait and will complete web-based questionnaires at baseline, just before the start of the training (3 months after baseline), and after completing the training (ie, approximately 6 months after baseline). Line managers’ direct reports will be invited to complete web-based questionnaires at baseline, 3 and 6 months later. Where available, data will also be collected from organizational records on the sickness absence levels of employees managed by the participating line managers.

### Measures for Line Managers (Intervention and Control)

The web-based questionnaire will contain the following items (Table 4). Demographics (including age, gender, number of years as a manager, and number of direct reports) were collected at baseline only.

**Table 4:** Summary of assessments.

Activity or measure			Enrollment	Baseline	On completion of intervention (approximately 6 weeks after baseline; intervention arm only)	3 months post baseline	6 months after baseline
Obtain enrollment data			✓				
Provide username and password			✓				
Web-based consent				✓			
**Manager only**							
	Demographics			✓			
	Confidence to create a mentally healthy workplace			✓	✓	✓	✓
	Mental Health Knowledge			✓	✓	✓	✓
	Workplace Mental Health Literacy			✓	✓	✓	✓
	**Management Competency Indicator Tool-manager version**			✓	✓	✓	✓
		WEMWBS^a^ 14		✓	✓	✓	✓
**Direct reports only**							
	Demographics			✓			
	WEMWBS^a^ 14			✓		✓	✓
	Management Competency Indicator Tool-employee version			✓		✓	✓
	Self-reported Productivity Rating			✓		✓	✓

^a^WEMWBS: Warwick-Edinburgh Mental Well-Being Scale.

Line managers’ confidence to create a psychologically healthy workplace will be quantified by a self-report measure used previously by Gayed et al [11,22] by modifying a previously published supervisor scale [31]. This measure includes 6 workplace scenarios (eg, “creating a work environment that prevents and reduces stress within my team”), which require line managers to rate their confidence on a scale of 1 (“not very confident”) to 5 (“very confident”). The items are summed to create a composite score with a range of 6 to 30, with higher scores indicative of more in creating a mentally healthy workplace.

The mental health knowledge schedule (MAKS) [23] will be used to measure the line manager’s mental health. It is a 6-item measure consisting of 6 key areas, such as help-seeking, recognition, support, employment, treatment, and recovery. MAKS is measured on a 5-point Likert scale ranging from 1 (“strongly disagree”) to 5 (“strongly agree”). Items are summed to create a composite score ranging from 6 to 30, with higher scores indicating better knowledge of mental health.

Workplace Mental Health Literacy [24] aims to measure line managers’ workplace literacy. It consists of 16 items, 4 vignettes featuring various manifestations of mental ill health within the workplace, and parallel questions exploring 4 dimensions of mental health literacy. These items are rated on a 5-point Likert scale ranging from 1 (“very low”) to 5 (“very high”). A higher score represents better workplace health literacy (range 16-80).

The SMCIT [13] measures management competencies surrounding the prevention and management of work-related stress. This measure consists of 36 items examining 4 key competencies: “managing emotions and having integrity,” “managing and communicating existing and future work,” “managing the individual within the team,” and “reasoning and managing difficult situations.” Items are measured on a scale of 1 (“strongly disagree”) to 5 (“strongly agree”). Each subscale is summed together, with higher scores indicating higher levels of competency. The measure has a version for both line managers (manager version) and employees (employee version), with the same content adjusted to suit the audience.

The WEMWBS [25] aims to quantify both line managers’ and employees’ psychological well-being over time. This measure consists of 14 items asking participants to rate a series of statements (eg, “I have been feeling useful”) on a scale of 1 (“none of the time”) to 5 (“all the time”). Summed scores range from 14 to 70 with higher scores representing better mental well-being.

After each of the training modules has been completed, participating managers will also be asked to complete a web-based feedback form, giving quantitative and qualitative feedback on the module. This will include questions on fidelity delivery and engagement with the training module [29]; questions on implementation qualities [29]; and open questions about how long the module took them to complete, which aspects of the module they found most useful, what they plan to change in their managerial practice, and how the module could be improved.

### Measures for Direct Reports (Intervention and Control)

#### Overview

On registering for the training, all participating line managers in both the intervention and control arms will be asked to send an invitation to participate in a web-based survey to their direct reports. The purpose of this is to assess whether completion of the training by line managers affects their direct reports’ perception of line managers’ behavior or the mental health and sickness absence outcomes of direct reports. The web-based survey for direct reports will contain the following items: demographics (age, gender, and number of years in the organization) collected at baseline only, WEMWBS 14-item [25], SMCIT-employee version [13], and a single-item self-reported productivity rating.

The web-based survey for direct reports at follow-up will contain the same items (as above) with the addition of open-response questions about any changes to how they are managed or their line manager’s behavior.

#### Organizational Sickness Absence Records

We will collect information from the organizations regarding the sickness absence records of those the participating line managers directly manage. Preintervention data will cover the 6 months before the intervention starts. Postintervention data will cover the 6 months after intervention completion. All organizational absence records will be anonymized at source before being securely transferred to the research team.

### Data Analysis

Statistical analysis will produce summary statistics to assess the parameters for a full trial. Missing data will be imputed using multiple imputations. SPSS (version 26; IBM Corp) will be used for statistical analysis. As this is a pilot trial, no emphasis will be put on the *P* values for any inferential statistical tests conducted. The primary analysis will use an intent-to-treat approach with repeated measures. Differences between the intervention and waiting list control groups at follow-up will be examined against baseline. The pilot data will provide information on the parameters needed for a realistic sample size calculation (mean, SD, and treatment effects of the primary outcome for the 2 arms) for a future main cluster RCT.

### Economic Measures

We will consider the feasibility of collecting data on costs and savings from the perspective of the employer. The costs considered will be the working hours lost to complete training during working hours, and, in turn, the cost or saving of any change in sickness absence rate over the follow-up will be calculated based on the median hourly wage (£15.6 [US $19.87] per hour) [32] of employees in the United Kingdom.

### Process Evaluation

#### Overview

A qualitative process evaluation will run alongside the pilot trial to explore the experience of the intervention with line managers and other stakeholders (eg, human resource managers and well-being coordinators) within participating organizations; assess the acceptability, usability, and utility of the intervention; and identify barriers and facilitators to implementation. Semistructured interviews will be used to collect the data to inform the process evaluation. In addition, module feedback forms will be completed by line managers after each of the 5 modules in the training course to allow more specific feedback relating to each module, including any changes in their managerial practice that participants are planning to make as a result of completing each module.

For the process evaluation interview study, participants will be invited to take part in interviews after the 6-month follow-up. Interviews will take place with (1) line managers in the intervention arm and (2) stakeholders in both the intervention and control arms.

Interviews will be conducted face-to-face, by video call, or by telephone at the participant’s preference and arranged at a mutually convenient time and location. The interview schedule will cover the following research questions and topics: acceptability, usability, and utility of the intervention; changes the line manager has introduced since undertaking the training; barriers and facilitators to the intervention implementation and effectiveness; and suggested improvements to the intervention. The interviews with stakeholders will cover the following research questions and topics: barriers and facilitators to taking part in the study; acceptability of the waitlist control condition (control arm only); acceptability, usability, and utility of the intervention; and barriers and facilitators to the intervention implementation and effectiveness. For stakeholders, the gatekeeper for each participating organization (intervention and control arms) will be asked to email an invitation to participate in an interview to all relevant stakeholders within their organization (eg, human resource managers, well-being managers, learning and development managers, and trade union representatives). Principles of data saturation will be used to guide the final sample size, but as a guideline, we would expect there to be interviews with approximately 20 line managers and 1-2 stakeholders per participating organization. All interviews and analyses will be conducted by members of the research team (CB, BV, and LT). Interviews will be guided by the interview schedules and audio recorded where consent is given. Digital audio files will be transcribed and fully anonymized.

#### Data Analysis

The qualitative data collected will be analyzed using thematic analysis [33,34] guided by the principles of framework analysis [35,36]. The Consolidated Framework for Implementation Research [37] and the Technology Acceptance Model [38,39] will be used to provide overarching frameworks for the qualitative analysis.

### Study Management

The study management group will be the research team based at the University of Nottingham and Nottinghamshire Healthcare National Health Service Foundation Trust’s Institute of Mental Health. A wider advisory group will include researchers from other UK universities who are part of the wider MHPP team and who will meet monthly to monitor progress. All data will be managed according to the GDPR, anonymized as soon as possible, and kept securely on a database that can only be accessed by the research team.

The study duration will be 24 months, with participating organizations starting to be enrolled in June 2021, and the study will cease at the end of May 2023.

### Criteria for Terminating the Study

The study may be stopped as a result of a formal or informal interim analysis and based on overwhelming evidence of efficacy or inefficacy, major safety concerns, new information, or issues with study conduct (eg, poor recruitment and loss of resources). The study may be stopped at a single site as a result of a formal or informal interim analysis and based on major safety concerns at that site, new information, or issues with study conduct at that site (eg, poor recruitment and loss of resources).

## Results

Recruitment for the study opened in September 2021 and closed in January 2022. A total of 24 organizations have consented to take part in the study, and 224 line managers have been recruited within these organizations. Data analysis was finished in August 2023, and we aim to publish the results later in 2024.

## Discussion

This protocol describes a feasibility trial that aims to assess the recruitment and retention rates for the study; the acceptability, usability, and utility of the MMW digital training intervention; the potential for improving outcomes for line managers and their direct reports; and the barriers and facilitators to the intervention implementation and effectiveness. This type of intervention fills an important gap as its primary focus is on the prevention of poor mental health at work through the design and management of work in a way that has been shown to promote mental well-being and prevent stress. Line managers have a critical role in this regard, and the initial testing of a digital intervention that is able to engage and be used effectively by line managers in a range of contexts is an important development. The results from this pilot and feasibility study will be a first step in informing the development of subsequent trials for MMW by providing insight into the usability and acceptability of the MMW digital training in a range of employment contexts and some initial indications of change in outcome variables for line managers and their direct reports.

We acknowledge that there are some limitations to the design to be adopted in this study. The intervention has been designed as general and relevant to line managers and supervisors across all sectors and contexts. Recruiting line managers from a wide variety of organizational contexts and sectors introduces a range of potential factors that could affect the delivery of the intervention (eg, work demands, reducing the time available to complete the intervention, and a lack of support from senior managers for mental health interventions), which could in turn affect the acceptability and usability of the results. However, the planned process evaluation interview data should help us identify some specific contextual factors that act as barriers to the intervention being accessed and used by participants. The use of a waitlist control group has been chosen to allow us to recruit more organizations and line managers into the pilot feasibility trial by offering the intervention to all participants by the end of the study. This design is often used in organization or workforce intervention studies due to the complexities of engaging businesses and public sector organizations in research and the ethics of ensuring control participants have the opportunity to potentially benefit from the intervention [40-42]. However, this is a limitation as it introduces possible biases. For example, we cannot rule out contamination of the waitlist control group. All organizations, both those allocated to the intervention and those in the waitlist control, were informed by the research team about the active components of the intervention to obtain organizational consent to participate in the study. Blinding organizations to their allocation to the intervention or waitlist control was not possible, as the research team had to explain the timing of the intervention delivery to the organizational stakeholders. To try to mitigate this, the research team ensured that organizations did not know who else was taking part in the study to avoid further contamination between sites. While we acknowledge the advantages of using single- and double-blinded designs to reduce such bias, this needs to be balanced with the pragmatism of conducting research in businesses and public sector environments.

## Abbreviations

GDPR: General Data Protection Regulations
MAKS: mental health knowledge schedule
MHPP: Mental Health and Productivity Pilots
MMW: Managing Minds at Work
RCT: randomized controlled trial
SMCIT: Stress Management Competency Indicator Tool
TiDiER: Template for Intervention Description and Replication
WEMWBS: Warwick-Edinburgh Mental Well-Being Scale

## Data Availability

The data sets generated during the study will be made available from the corresponding authors upon reasonable request.
